# New people, new policy: How personnel renewal in the party executive affects party policy change. The case of Austria

**DOI:** 10.1177/13540688241293052

**Published:** 2024-10-21

**Authors:** Matthias Kaltenegger, Wolfgang C Müller

**Affiliations:** 27258 University of Vienna, Austria

**Keywords:** intra-party politics, party change, party executive, party organization, party policy

## Abstract

The party organization literature has long acknowledged that changes in a party’s internal power structure bring about changes in leadership personnel. However, empirical assessments of how such personnel changes relate to party behavior are rare. We explore personnel renewal in the party executive as a driver of party policy change and argue that focusing on these processes provides for an encompassing perspective on the connection between intra-party power and party behavior. Building on the party change literature, we theorize on personnel renewal in the party executive as a potential stand-alone driver of party policy change as well as on its interplay with other explanatory factors and test empirical implications based on all major Austrian parties (1949–2019). Findings suggest that personnel renewal in the party executive has a profound effect on party policy and that these personnel changes are in part driven by party performance.

## Introduction

Why do political parties change their policy platforms and the salience they attribute to policy domains in particular? A rich literature has provided a myriad of valuable insights on the various factors driving party policy change (e.g. Abou-Chadi et al., 2020; Adams et al., 2004; Fagerholm, 2016; Harmel et al., 1995; Klüver, 2020; Meyer, 2013; Panebianco, 1988; Somer-Topcu, 2009; specifically on the parties’ policy agendas, e.g. Green-Pedersen, 2019; Gunderson, 2024; Klüver and Spoon, 2014; Kriesi et al., 2012; Meyer and Wagner, 2019).

However, important aspects of these processes are still relatively understudied. This particularly applies to the role of intra-party politics. While theories of party change identify shifts in parties’ internal power configurations as potential driver of party change (Harmel and Janda, 1994; Panebianco, 1988), empirical studies focusing on the topic are still relatively scarce. Extant contributions engaging with this question largely conceive intra-party power as a static structural characteristic of the party, thus drawing inferences on the effects of intra-party power based on variance between parties (Abou-Chadi and Orlowski, 2016; Hennl and Franzmann, 2017; Koedam, 2022b; Lehrer, 2012; Marini, 2023; Meyer, 2013; Schumacher et al., 2013). Only a few studies have begun to unravel the effects that within-party variations in intra-party power distribution have on party policy change (Ceron, 2012; Ceron and Greene, 2019; Kaltenegger et al., 2021).

With this paper, we seek to contribute to this literature. We propose that personnel changes in party executive bodies are important to understand how intra-party politics affects party change. While previous studies have focused on the power distribution between intra-party groups – what Panebianco (1988) dubbed the ‘*conformation*’-dimension of intra-party coalitions – we provide the first systematic account on how change in their ‘*composition*’ – Panebianco’s second dimension – relates to party behavior. In so doing, we follow the rather simple intuition that ‘personnel is policy’ (Lewis, 2008): We explore to what extent personnel renewal in executive bodies triggers party policy change and in what ways these processes interact with other drivers of party change.

We focus on the party executive as these bodies have a key role in making party policy in most parties (Poguntke et al., 2016). While democratic parties typically assign supreme power to the party congress, this body meets infrequently and it is largely the party executive which sets the congress’ agenda and steers its proceedings (Aylott and Bolin, 2017; Kaltenegger et al., 2021; Ware, 1986). Between the congresses, the executive is the supreme authority for making party policy. Clearly, the party leader is likely to provide leadership in this regard and may even play a dominant role within the executive. However, unlike most congress delegates, members of the party executive are professional politicians who interact on a regular basis and who are endowed with their own power bases within the party. Even with a strong party leader, the executive body thus retains veto power over party policy.^1^ Changing party policy hence requires at least the toleration if not active support by the party executive. In this vein, we consider personnel changes in the party executive a potential driver of party policy change, which has so far been largely neglected in the literature (though see Müller et al., 1992; Schonfeld, 1981).

Empirically, we study a) whether changes in the composition of the main parties’ national executive bodies are associated with changes in the salience the parties attach to policy domains in their electoral manifestos and b) whether these personnel changes mediate the impact of other drivers of party policy change. Drawing on data on the main Austrian parties for the entire post-war period, we find our main expectation confirmed that personal renewal of the party executives is associated with change in party policy.

## Party organization and party policy change

Much of the political science literature has generally conceived party change as the strategic reactions of party leaders to external pressures (Budge, 1994; Downs, 1957). Following this line of reasoning, parties have been found to adapt their policy platforms to swings in public opinion (Adams et al., 2004), to changing economic conditions (Haupt, 2010; Ward et al., 2011), to other parties’ positional shifts (Adams and Somer-Topcu, 2009) or when experiencing electoral defeat (Somer-Topcu, 2009; Walgrave and Nuytemans, 2009). In these ways, parties – as collective entities – react ‘rationally’ to changes in their competitive environments. Their specific reactions to different environmental stimuli, however, have been found to depend on different party characteristics. These include the parties’ government/opposition status (Meyer, 2013; Schumacher et al., 2013; Walgrave and Nuytemans, 2009), their ‘nicheness’ (Ezrow et al., 2011) and resources (Wagner and Meyer, 2014). Closest to our perspective, some studies focus on the parties’ decision-making structures as particularly influential moderators (Abou-Chadi and Orlowski, 2016; Hennl and Franzmann, 2017; Lehrer, 2012; Marini, 2023; Meyer, 2013; Schumacher et al., 2013). Collectively, these studies demonstrate that the distribution of intra-party power indeed has substantial implications for party behavior, as parties with more centralized decision-making structures are more likely to change policy stances.

A notable limitation of this strand of the literature, however, is its rather static perspective on party organizations. Particularly, power structures are predominantly inferred from party rules or expert assessments and conceived as a relatively stable feature of the organization. Shifts in intra-party power relations, where the influence of specific actors in decision making increases at the cost of other actors’ influence, are thus largely unaccounted for. This is somewhat surprising, considering that such processes are at the core of what theories of party change (Harmel and Janda, 1994; Panebianco, 1988) have conceptualized as the ‘internal causes’ of change. Hence, aside from qualitative case studies and a few notable exceptions in the quantitative literature (Ceron, 2012; Ceron and Greene, 2019; Giannetti and Laver, 2008), there exists somewhat of a mismatch between how theoretical accounts and how the bulk of the empirical literature have approached the implications of intra-party politics for party change.

## The internal drivers of party policy change

In Harmel and Janda’s (1994: 265) seminal framework, the party-internal processes of change in who is the party leader and change in what is the dominant faction are considered as independent drivers of party change, which at times interact with exogenous ‘shocks’. Yet, there is little empirical work engaging with the actual effects of intra-party dynamics on party change. A few studies accounting for change of the party leader provide no clear support for the expectation that new leaders (alone) drive party policy change (Bille, 1997; Harmel et al., 1995; Meyer, 2013).^2^ Similarly, evidence for the impact of factional power shifts on party policy is mixed, yet there is substantial uncertainty attached to some of the extant studies due to data limitations. Some comparative studies have employed qualitative assessments of factional power shifts, reporting tentatively that the effects of these processes may be conditional on other factors (Harmel et al., 1995; Harmel and Tan, 2003). However, more recent contributions, using factional motions at party congresses, demonstrate quite clearly that the relative power of party factions matters for party policy (Ceron, 2012; Ceron and Greene, 2019; Giannetti and Laver, 2008).

## Intra-party power and the composition of party executive bodies

The empirical studies cited above have focused on party factions to identify change in the distribution of intra-party power, thus accounting for horizontal intra-party divides with at least a minimum level of stable organizational structure. Arguably, however, the reality of intra-party competition is more complex, and factionalism (in this sense) is only one form of intra-party competition, which may affect party behavior. While conceptualizations vary throughout the literature (e.g. Boucek, 2009; Dilling, 2024; Duverger, 1954; Kölln and Polk, 2024; Panebianco, 1988; Rose, 1964), we may broadly differentiate between the following divides in intra-party competition (see Table 1): 1. *factions* (Boucek, 2009; Ceron, 2019; Rose, 1964), 2. *tendencies* (Rose, 1964), 3. *single-issue pressure groups* (Bale, 2023), 4. *hierarchy* (Bäckersten, 2022; Duverger, 1954; Kitschelt, 1989; May, 1973; Müller and Strøm, 1999; Norris, 1995), 5. *components/’faces’ of the party* (Hagevi, 2018; Katz and Mair, 1993), 6. *personal networks/cliques* (Duverger, 1954: 152; Martínez-Cantó and Verge, 2023), 7. *generational divides* (Panebianco, 1988: 242).

**Table 1.** Types of intra-party divides.

*1. Factions*	Permanently organized intra-party groups, representing specific interests (Boucek, 2009) such as occupational groups (e.g. union groups), territorial sub-units (e.g. regions) or specific ideological currents (Ceron, 2019; Rose, 1964).
*2. Tendencies*	Loose and fluctuating but durable groups, lacking permanent organizational structures (Rose, 1964). Adherents of a tendency share a common interest such as ideological or party organizational principles. There are no clear criteria for tendency membership other than the members’ identification with the tendency’s cause.
*3. Single-issue pressure groups*	Support groups of a common short-term goal, for instance, a specific policy or a particular party organizational reform measure (e.g. ‘ginger groups’ in the British Conservative Party) (Bale, 2023) which typically dissolve after intra-party competition over their common goal has resumed.
*4. Hierarchy*	‘Vertical’ divides between the different levels of the organizational hierarchy (Duverger, 1954; Kitschelt, 1989; May, 1973; Norris, 1995) that are typically rooted in differences in goal priorities – e.g. office-oriented party elites and policy-oriented rank-and-file members (Bäckersten, 2022; Kaltenegger et al., 2021; Müller and Strøm, 1999; Strøm, 1990) – and likely to be activated when the party faces tradeoffs between these party goals or when a reform of intra-party decision-making structures (and thus the relative influence of each level on party decisions) is up for debate.
*5. Components/'faces’ of the party*	Divisions between the ‘party on the ground’ (members, activists), the ‘party in central office’ (organizational bureaucracy) and the ‘party in public office’ (Katz and Mair, 1993) which are rooted in different goal priorities and incentive structures (Hagevi, 2018).
*6. Personal networks*	Loosely organized and relatively small groups of co-partisans teaming up for their common career benefit (e.g. supporting each other in party nomination or intra-party election processes) (Duverger, 1954: 152), often operating discretely.
*7. Generational divides*	Different cohorts of party politicians are likely to hold different sets of ideas and preferences (Jennings and Niemi, 1981; Panebianco, 1988: 242; van der Brug and Kritzinger, 2012) with the future having a higher value for the newer generations (e.g. issues of climate change, pension systems). At times, latent patterns of generational disagreement develop into a salient intra-party conflict dimension (Müller and Meth-Cohn, 1991).

These different divides may exist in any party simultaneously, they typically overlap in various ways, and they may be more or less salient in different situations depending on party organizational and contextual factors. Considering this multidimensionality in intra-party competition it may be problematic that the literature has so far almost exclusively focused on one divide (type 1). This approach a priori disregards the potential effects of other forms of intra-party competition and thus potentially underestimates the broader significance of party-internal power dynamics for party change (especially for parties with low levels of factionalism or even for factionalized parties, in periods where other intra-party divides are more salient). Intra-party power shifts across any divide have the potential to impact party behavior and there is no reason to suspect that factional politics are per se more consequential than other types. Yet, once we aim at a more encompassing perspective on intra-party competition, identifying the relevant intra-party divisions in specific situations and subsequently measuring power shifts presents us with a challenge: While the behavior of party factions is to some extent observable for researchers due to their relative stability and their formal organizational structures, other intra-party groupings are more short-lived (e.g. types 3, 6), they have little or no formal organizational structure (e.g. types 2, 3, 6, 7) and they sometimes deliberately operate in secret (e.g. type 6). Even when these measurement issues may be resolved conceptually, it is unlikely that all divides – let alone their interactions – can be accounted for empirically.

To address this problem, we propose that studying personnel changes in the party executive – while abstracting from specific intra-party divides – offers a useful, ‘global’ perspective on change in intra-party power. We thus shift the empirical focus from what Panebianco (1988: 39) labeled the *conformation* of a dominant coalition to its *composition*. Whereas conformation pertains to the distribution of power, the cohesion and the stability of a party’s dominant coalition, composition refers to the specific people forming the coalition. As indicated by various qualitative case studies (e.g. Luther, 2011; Massey, 2021; McKenzie, 1982), changes in conformation (e.g. the power balance between intra-party groupings) naturally go hand in hand with significant personnel changes in key party offices (Harmel and Janda, 1994). Intra-party power shifts of any kind are thus generally reflected in the amount of change in the composition of party executive bodies.

To be sure, some compositional change in decision-making bodies will always be driven by routine replacements of party veterans, due to biological or – in parties with age or term limits for party office – statutory necessity. While these changes may or may not relate to the divides 1 to 6, they are part of the incremental process of generational change (type 7). Shifts across horizontal intra-party divides (types 1 to 3) most likely follow a roughly Gamsonian logic (Gamson, 1961), with specific groups attaining control over party executive seats in approximate proportion to their command over decisive resources such as party members or voter support in elections (Ceron, 2012, 2014; Ennser-Jedenastik, 2013). Likewise, change in the power equilibrium between the party elite and the rank and file (type 4) will affect to what extent the party elite may contain change in the composition of their group (e.g. to secure the personal benefits tied to their role), or conversely, to what extent the rank and file may hold party executive members accountable through deselection (Andrews and Jackman, 2008; Bynander and t’Hart, 2007; Ennser-Jedenastik and Müller, 2015; Ennser-Jedenastik and Schumacher, 2021). Similarly, power relations between the party in central office and the party in public office (type 5) will be reflected in the latter’s ability to ‘colonize’ the party executive (Blondel, 2000). Finally, personal networks between party elite actors (type 6) can be expected to factor into the composition of the party executive in various ways, for instance, when a new party leader seeks to establish a stable support group of trusted individuals within the party executive (Martínez-Cantó and Verge, 2023).

In this vein, we conceive change in the composition of party executive bodies as a form of party-internal change, encompassing any variation in the specific set of party decision makers. Depending on the magnitude of change in intra-party power relations, this variation may range from routine replacements of single members to extensive personnel renewal of the leadership body. While unspecific in terms of tracing the relative power of specific intra-party groups in certain situations, this approach is more encompassing. It accounts for all types of power swings within the party, even when the specific intra-party groups and group members involved are unknown to the researcher.

## Party executive renewal, external pressures and party policy change

How does change in the composition of the party executive fit into theoretical models of party change? Notwithstanding the idiosyncratic reasons for specific personnel changes in party executives, they put new decision makers in charge, thus interfering with the organization’s internal power structure (Lewis, 2008; Massey, 2021; Panebianco, 1988: 242). Each replacement in the party executive creates opportunities for party change, as each individual can be expected to insert new concerns and ideas due to his or her personal background or pattern of affiliations with intra-party groups. Analogous to previous conceptualizations of the internal causes of party change (Harmel and Janda, 1994; Panebianco, 1988), we therefore expect that greater amounts of change in composition should lead to higher levels of party policy change.

Hypothesis 1 The more personnel renewal in the party executive, the greater party policy change.

The theoretical literature sets great store on external shocks as drivers of party change and on their interaction with internal drivers (Harmel and Janda, 1994; Panebianco, 1988: 242). We test these expectations focusing on shocks relating to the party’s performance in competition, specifically its electoral performance and its access to government office. Underperformance in both puts pressure on a party to change (Bale, 2012; Gauja, 2016; Greene and Haber, 2016; Harmel and Janda, 1994; Scarrow, 2015). While unspecific on their connection with party-internal drivers, theoretical accounts hint at the possibility that the effects of such external pressures on party change may be mediated by changes in the party’s internal power structure (Harmel and Janda, 1994: 267). Indeed, it is even likely – and in line with work on party leader survival (Andrews and Jackman, 2008; Bynander and t’Hart, 2007; Ennser-Jedenastik and Müller, 2015; Ennser-Jedenastik and Schumacher, 2021) – that changes in party leadership often result from exposure to external pressures: Unsuccessful incumbent leaders are held accountable for the party’s underperformance. Their replacement by challengers, who are more willing to change the party’s course, may then be the mechanism by which a party adapts to its environment. Hence, personnel changes in the party executive are a likely mediator through which competitive pressures impact party policy.

Hypothesis 2a The effect of electoral performance on party policy change is mediated by personnel renewal in the party executive.

Hypothesis 2b The effect of office loss on party policy change is mediated by personnel renewal in the party executive.

**Figure 1.**
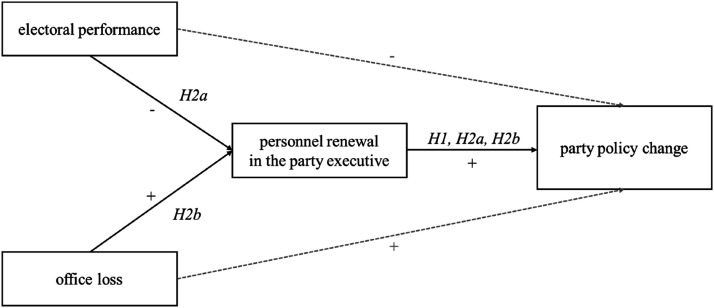
Hypothesized relationship between competitive pressures, personnel renewal in the party executive and party policy change.

As we hypothesize that a party’s past performance will influence changes in its policy platform – at least partially – through personnel renewal in its leadership bodies, our observational analysis should reveal the following pattern (Baron and Kenny, 1986): Underperformance in terms of votes and office increases personnel renewal in the party executive, which subsequently drives changes in party policy (see Figure 1). Finally, electoral performance and office loss should impact party policy change when omitting personnel renewal from the analysis.

## Empirical strategy

To test these hypotheses, we examine policy change of the major Austrian parties in the 1949–2019 period, focusing on the relative importance parties attach to policy domains. Austria resembles many other party democracies, where government is provided through and by the parties, regarding core characteristics of the party system and of party competition. The major parties have remained the same over the investigation period, although their electoral and, even more so, office fortunes have varied considerably. While new parties emerged, only the Greens and NEOS have become permanent additions to the party system. Compared to other European parliamentary democracies, Austria has historically been characterized by a higher level of party system stability and by a lower rate of alternation in the (predominantly coalition-format) government. Particularly the latter aspect limits the generalizability of our findings regarding H2b.

Organizationally, Austrian parties are comparatively strong in terms of membership, geographical representation and finance. Despite ideological differences and some organizational specifics (e.g. Heinisch, 2016), the parties share commitment to a system of intra-party democracy that is based on a regime of delegation and accountability via party bodies rather than the direct voice of members (Jenny, 2018). Among party bodies, the executives have a strong position and are the main source of authority within the party at large, formally subordinated only to the party congress. In terms of party policy, the party executives enjoy superiority vis-à-vis the parliamentary party, making the direction-setting decisions such as those on electoral manifestos but leaving their daily realization to the parliamentary parties (Dolezal et al., 2012). The statutory powers of the party executives have largely remained unchanged in our observation period (Müller, 1992; Poguntke et al., 2016). As highly institutionalized and long-living organizations, the main Austrian parties constitute a good case for investigating our research question. To the extent that other parties share their organizational characteristics, our findings should be generalizable within the universe of Western European parties. Due to its characteristics, Austria might be a more likely case to finding a positive relationship between personal renewal of the party executive and party policy change. Hence, if we do not find it there, it would be quite unlikely to find it anywhere else.

In the empirical analysis, we utilize change in issue emphasis (salience) as our measure of party policy change. While saliency theory generally provides important insights into key dynamics of inter-party competition (Budge, 1994; Budge and Farlie, 1983; Dolezal et al., 2014), intra-party competition is particularly likely to affect the relative emphasis the party puts on different policy issues (Green-Pedersen, 2019; Gunderson, 2024; Meyer and Wagner, 2019; Wagner and Meyer, 2014). We derive our measure for change in issue emphasis from hand-coded manifesto data, following the bulk of the extant literature.^3^ We use all electoral manifestos of the five currently relevant parties (SPÖ, ÖVP, FPÖ, Greens, NEOS; 1945–2019) and record changes in issue emphasis across 20 issue areas based on the AUTNES hand-coding scheme (Dolezal et al., 2016; Müller et al., 2012). Specifically, we first allocate each of the 680+ AUTNES issue categories to one of 20 broader categories: *welfare (services), taxes, labour, capital, regulation, security, social values, multiculturalism, education, environment, urban/rural, Europe, foreign policy, defense, constitutional issues, infrastructure, protest, ideology, government formation*, and a *residual* category (Dolezal et al., 2014). We then measure each area’s salience in a given manifesto using the log measure of issue emphasis suggested by Lowe et al. (2011).^4^ Finally, we calculate a party’s overall change in issue emphasis between *t*^*−1*^ and *t* by aggregating the changes in salience across all issue areas and dividing this value by two (see Supplemental Appendix).

To measure personnel renewal in the party executive, we build on a comprehensive dataset on party-internal elections at Austrian party congresses. These data comprise information on the composition of party executive bodies in all major parties since 1945, thus allowing to track changes over time. We further complement these data with information on ex-officio (e.g. incumbents of high public office) party executive members. We then generate our renewal measure by calculating the percentage of party executive members at time *t* (e.g. the last party congress before the national election), who have not been part of the party executive at time *t*^−1^ (e.g. the last party congress before the previous national election) (see François and Grossman, 2015).^5^ In this way, our measure simply records the share of new people within the party executive relative to the cohort involved in drafting the last manifesto.

To account for competitive pressures, we include measures for vote change (in percent) (Adams et al., 2004; Adams and Somer-Topcu, 2009; Schumacher et al., 2013) and for the loss of government participation (‘0’/‘1’) at the previous election (Ennser-Jedenastik and Schumacher, 2021) in our regression models. We further control for change in who is the party leader in all models (‘1’ if a party has experienced a change in its leader since the last national election, otherwise ‘0’). Leadership change is the second potential party-internal driver of party change, which can be expected to affect other personnel changes in the party executive as well as the party’s policy platform (Harmel and Janda, 1994). We additionally control for parties’ government status when predicting change in issue emphasis, as government parties have been found to be more prone to policy change than opposition parties are (Meyer, 2013; Schumacher et al., 2013). Finally, we include party and 20-year-period fixed effects in all regression models to account for systematic differences between parties due to party organizational characteristics (e.g. party size, party rules) and for periodic patterns. Additional information on key variables and robustness checks are provided in the Supplemental Appendix.

## Analysis

**Figure 2.**
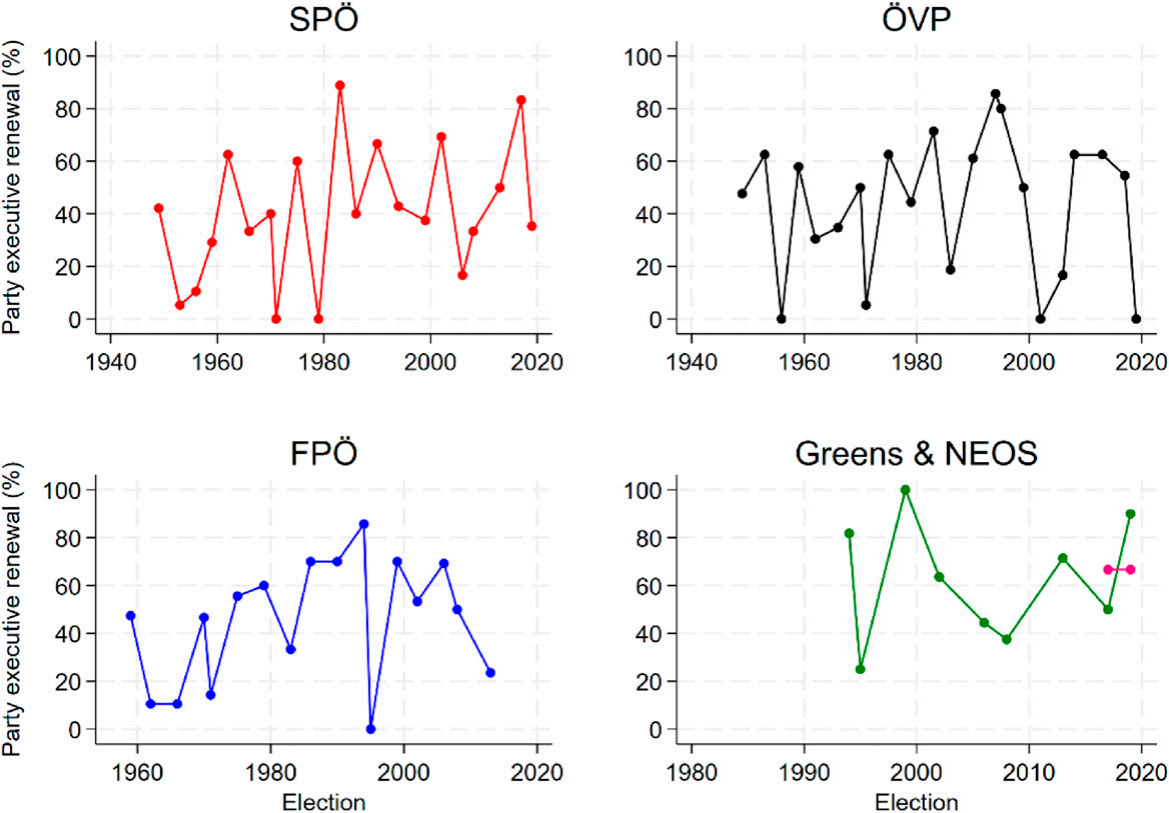
Party executive renewal (%) in Austrian parties.

Before we turn to multi-variate analysis, Figure 2 provides an overview of party executive renewal (by party), revealing considerable variation over time in all parties (Figure 2). By tendency, this supports our perspective that these personnel changes are not a mere mechanical process of steady generational replacement, but that they rather reflect broader intra-party dynamics. Note that the number of members in the executive bodies studied varies between parties and over time, ranging from 5 to 24 (see Supplemental Appendix).

**Figure 3.**
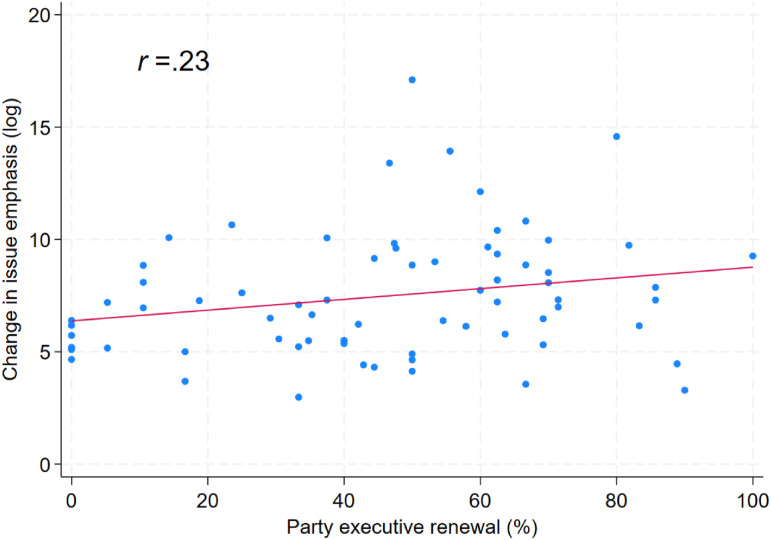
Correlation of party executive renewal and change in issue emphasis.

Figure 3 further displays a two-dimensional mapping of changes in the composition of the party executive and changes in issue emphasis. Given that Austrian manifestos cover the whole range of policy dimensions, there is a natural cap on changes in issue emphasis while party executive renewal ranges from 0 to 100%. While our two key variables are positively correlated, as expected, their bivariate association is modest (*r*=.23, *p* < 0.1) and the extremes on both dimensions are typically not associated with extremes on the other.

**Figure 4.**
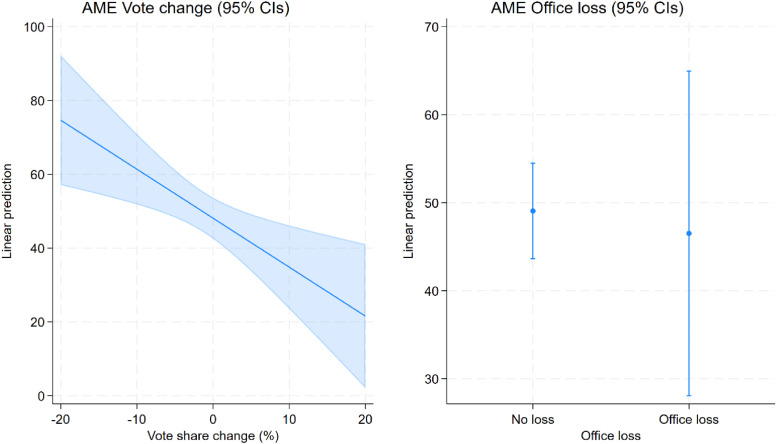
Average marginal effects of vote change (H2a) and office loss (H2b) on party executive renewal; Model 1.

We proceed to regression analyses for a more formal test of our hypotheses (Table 2). We first examine whether party executive renewal is driven by a party’s past electoral performance and by losing government office – thus following the sequential logic underlying our theoretical reasoning on the connection between competitive pressures, personnel renewal and party policy change (Figure 1). Accordingly, we use personnel renewal in the party executive as the dependent variable, clustering standard errors by year and accounting for party as well as period fixed effects (Model 1). Expecting a correlation between personnel renewal in the party executive and leadership change, we also control for the latter. Results indicate that a party’s performance in the last election indeed significantly and substantively affects change in the composition of the party executive. A one percent gain in the party’s vote share decreases personnel renewal in the party executive by approximately 1.3%, which roughly amounts to a 40% difference in renewal rates across the empirical range of the vote change variable (Figure 4). This suggests that party executive members are held accountable for the party’s electoral performance, as expected, whereby the risk of being replaced diminishes the more the party gains at the polls. Contrarily, however, we find no support for an effect of office loss on personnel renewal (Figure 4). Hence, the mediation condition requiring the independent variable to influence the mediator is met for electoral performance (H2a), but not for office loss (H2b). Perhaps losing office is a particularly shocking experience to parties, especially to those accustomed to governing, often leading to the party leader’s resignation. In such situations, it may be the natural reaction to close the ranks and provide stability in the collective leadership. There may also be less competition for these positions in a fresh opposition party.

**Table 2.** OLS regressions on party executive renewal (Model 1) and on change in issue emphasis (Models 2–4); standard errors clustered by year (Model 1) and by election (Models 2–4).

	DV: Party executive renewal (%)	DV: Change in issue emphasis (log)		
	(1)	(2)	(3)	(4)
Party executive renewal (%)		0.0268**	0.0317**	
		(0.00931)	(0.0110)	
Leadership change	5.641		−0.373	−0.188
	(5.617)		(0.675)	(0.651)
Vote share change (%)	−1.327**		0.0207	−0.0426
	(0.452)		(0.0523)	(0.0617)
Office loss	−2.556		−1.496	−1.405
	(9.334)		(1.037)	(1.040)
Party in government			−0.284	0.00158
			(0.716)	(0.693)
Party FE	yes	yes	yes	yes
20y-period FE	yes	yes	yes	yes
Constant	22.95*	5.158***	5.417***	5.934***
	(10.15)	(0.477)	(0.871)	(0.772)
N	65	71	69	73
R^2^	0.355	0.312	0.332	0.269

Standard errors in parentheses; + *p* < 0.10, *p < 0.05, **p < 0.01, ***p < 0.001.

**Figure 5.**
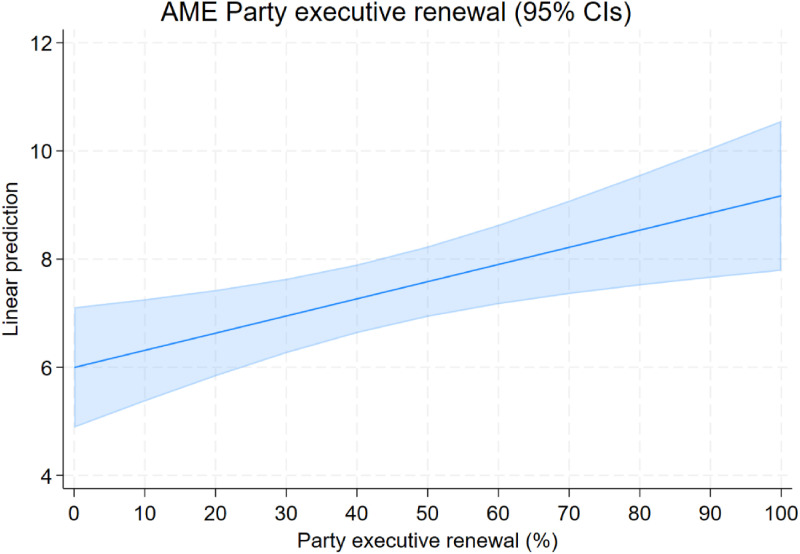
Average marginal effects of party executive renewal (%) on change in issue emphasis (H1); Model 3.

Next, we turn to the direct effect of our main variable of interest, personnel renewal in the party executive, on party policy change (H1). To this end, we run OLS regressions using the amount of change in a party’s issue emphasis as the dependent variable and clustering standard errors by election. Results clearly support H1. As expected, party executive renewal has a positive and statistically significant effect on change in issue emphasis in Models 2 and 3. This effect holds substantive significance as well. Moving from 0 to 100% renewal in the party executive increases change in issue emphasis by approximately one standard deviation (Figure 5). Thus, the more party executive members have been replaced since the last manifesto was drafted, the more pronounced the shifts in issue emphasis. Naturally, this also means that the mediation condition requiring the mediator to affect the dependent variable is fully met (H2a, H2b).

However, testing the final mediation condition, that the independent variables affect our dependent variable when excluding the mediator from the estimation, we find no statistically significant effects of a party’s past electoral performance and of losing government office on change in issue emphasis (Model 4). Hence, this condition for mediation is neither met for vote change (H2a) nor for office loss (H2b).

Overall, there is no indication for a mediation of office loss by party executive renewal (H2b). However, given the limited variation in the office loss variable and our number of observations, we may lack the statistical power to detect effects of smaller size. Regarding the mediation of electoral performance (H2a) our findings are mixed. Our analysis suggests that a party’s electoral underperformance indeed drives personnel renewal and that these changes in the party’s leadership personnel subsequently impact party policy. Yet, as we do not find a significant direct effect of vote change on the dependent variable, only two out of three conditions for mediation are met based on our small sample. This may again be related to idiosyncrasies of the case, such as the relatively secure access to government for the two largest parties and the consistently low levels of electoral volatility throughout much of the observation period.

## Conclusion

In this paper we advance a very simple intuition: new people in the main party executive body are likely to introduce party policy change. Based on comprehensive data on the composition of party executives in Austria (1949–2019), we analyze how personnel renewal in these decision-making bodies affects change in issue emphasis.

By focusing on the composition (e.g. the specific people forming the group of party decision-makers) rather than the conformation of intra-party coalitions (e.g. the relative strength of specific intra-party groups) (Panebianco, 1988), we propose a novel perspective on change in intra-party power. This is an encompassing approach, that incorporates power-shifts along various divides in intra-party competition, which are all potentially consequential for party behavior, but often extremely difficult to study systematically. By shifting the focus from intra-party groups to individuals, we contribute an empirically straightforward and substantively ‘holistic’ perspective on how intra-party politics affect party behavior.

The empirical analysis supports our expectation that party executive renewal drives party policy change. Specifically, the more ‘new faces’ gain access to the party executive, the more likely the party will change its relative focus on policy issues. This result demonstrates quite clearly that changes in a party’s leadership personnel matter for party policy. The fact that we find these effects consistently – while finding no evidence for effects of other, theoretically more established potential drivers of party change – further underscores the significance of these processes for party behavior.

Based on our observational data, we also investigate whether personnel renewal in the party executive operates as a mediator through which external pressures affect party change (Harmel et al., 1995; Harmel and Janda, 1994). While results on mediation are inconclusive overall, we find that a party’s electoral performance significantly influences the extent of personnel change within a party’s leadership body, which subsequently drives shifts in issue emphasis.

To be sure, our findings on the null effects of competitive pressures and – by extension – the largely inconclusive evidence on mediation must be taken with a grain of salt considering some evident limitations of our study. Although we cover all currently major parties over an extensive observation period, we still analyze just one country, and we still have a relatively small number of observations overall. Due to characteristics of the Austrian political system, we are further constrained by limited variation for some of our explanatory variables (particularly loss of government office), which consequently restricts statistical power. Not least, while we employ the simple causal steps approach (Baron and Kenny, 1986) due to these data-related constraints, this approach has been contested in more recent methodological accounts on mediation analysis (Hayes, 2022).

Hence, we perceive the empirical analysis presented in this paper as a beginning rather than an end. Our data are well suited for a first exploration of our primary research interest – the effects of personnel change on party policy – and have enabled us to establish party executive renewal as a consequential manifestation of intra-party power dynamics for the case studied. We are also confident that this key result will travel beyond the ‘likely’ Austrian case to other countries with strong and relatively centralized parties. However, a comparative perspective is still needed to assess the broader generalizability of our findings, particularly of those pertaining to the interplay of party-internal and party-external drivers of change. Making a substantiated causal claim in this regard would further require applying alternative methodological approaches (e.g. process tracing). Moreover, we believe that future research should engage in more detail with the connection between the conformation and the composition of intra-party coalitions and explore more specifically to what extent (and under what conditions) power shifts across different divides affect changes in leadership personnel.

## Supplemental Material

**Supplemental Material -** New people, new policy: How personnel renewal in the party executive affects party policy change. The case of Austria Supplemental Material for New people, new policy: How personnel renewal in the party executive affects party policy change. The case of Austria by Matthias Kaltenegger and Wolfgang C Müller in Party Politics.

## Notes

1 Occasionally, party leaders were granted special powers (most recently, ÖVP leader Sebastian Kurz), but these episodes quickly ended when the leader’s electoral or political fortunes vanished.

2 Note, however, that case studies on particular instances of leadership change indicate pronounced effects for these cases (e.g. Minkin, 2014).

3 Party manifestos are not a perfect reflection of the policies parties implement, as they often involve strategic considerations and as parties face various constraints when translating agendas into action. However, extant research indicates that parties, in Austria and elsewhere, indeed pursue and implement a substantial share of the policies they propose in their manifestos (Praprotnik, 2017; Thomson et al., 2017). Not least, for a long observation period such as ours, party manifestos are the only systematic data source to construct measures of issue emphasis.

4 For other, yet, similar approaches to measuring issue emphasis and change thereof see Koedam (2022a) and Marini (2023).

5 Note that we exclude the party leader when calculating party executive renewal, as leadership change is considered using a separate variable in the analysis (see Supplemental Appendix).
