# Supplementary material for: New people, new policy: How personnel renewal in the party executive affects party policy change. The case of Austria
Source: Party Politics. 2024 Oct 21;31(6):952–64. doi: 10.1177/13540688241293052 (PMC12533963; doi:10.1177/13540688241293052)
Supplement: Supplemental Material - New people, new policy: How personnel renewal in the party executive affects party policy change. The case of Austria [file sj-pdf-1-ppq-10.1177_13540688241293052.pdf]

Appendix:

New People, New Policy: How Personnel Renewal in the Party Executive Affects Party Policy Change

The Case of Austria

Descriptive Statistics

Table A1: Descriptive statistics of key variables.

	Mean	Sd.	Min.	Max.
Change in issue emphasis (log)	7.548912	2.695547	2.979101	17.10428
Party executive renewal (%)	46.09228	26.04836	0	100
Leadership change	.4487179	.5005824	0	1
Vote share change (%)	-.1616438	4.689979	-16.89	15.4
Office loss	.0821918	.2765574	0	1

Overview: Party Executive Renewal

Table A2: Party executive name and number of members (by party and period).

	Years	Executive body (name)	Mean	Min.	Max.
SPÖ	1945–1956	Parteivorstand	19	19	19
	1956–1967	Parteiexekutive	24	24	24
	1967–2023	Bundesparteipräsidium	9.3	5	18
ÖVP	1946–1948	Bundesparteivorstand	15	15	15
	1948–1951	Bundesparteipräsidium	21	21	21
	1951–1989	Bundesparteivorstand	15.4	8	23
	1989–2015	Bundesparteipräsidium	8.5	5	18
	2015–2023	Bundesparteivorstand	22	22	22
FPÖ	1956–1976	Bundesparteivorstand	16.6	12	19
	1976–2023	Bundesparteipräsidium	12.7	5	20
Greens	1986–2023	Bundesvorstand	8.8	7	11
NEOS	2012–2023	Vorstand	7.3	6	10

Note: The party leaders are excluded from these counts for analytical reasons.

All parties studied have more than one executive body. Typically, a smaller ('inner') executive consisting of the party leader and a handful of party heavy-weights (approx. 5–25 members) forms the core of one or two more inclusive (and thus larger) executive bodies. These structures have also evolved over time. We account for this by constructing our measure of party executive renewal focusing on the most exclusive – and plausibly, the most

influential – party executive body in each party at each point in time according to the party statute. Due to changes in party rules and other factors (e.g. the party's control over public offices granting its holders ex-officio membership in the party executive), the number of party executive members as well as the names of the relevant executive bodies vary over time. Table A2 lists the specific executive bodies we focused on for each party and period including summary statistics on the number of executive members.

The composition of party executive bodies, our main independent variable, is a largely neglected topic of research. It thus merits to provide a brief descriptive inspection. Figure 2 in the main paper and Figure A2 display the percentages of personnel renewal across parties and over time. While the mean renewal rate is relatively similar for SPÖ, ÖVP and FPÖ with values between 40 and 45 percent, the Greens and NEOS record averages of over 60 percent, respectively. Likewise, while renewal below 25 percent is not particularly rare for the three larger parties (with all of them even recording instances of zero renewal), both the Greens and NEOS have consistently surpassed this threshold. Intuitively, these party differences mirror extant ratings of intra-party democracy in Austrian parties, with more internally democratic parties experiencing more change in the composition of the party executive overall (Bolin et al., 2017; Müller et al., 1992; Müller and Meth-Cohn, 1991).[1] Bivariate inspections further reveal that personnel renewal correlates negatively with the party's past electoral performance (*r*=-.26*) and that it is over 20 percent higher when coinciding with leadership change (*diff*=20**). This is also reflected in the regression results displayed in Table A5. While a change in who is the party leader increases personnel renewal in the party executive in Model 9, this effect disappears when accounting for vote change, which retains its negative and significant effect throughout.[2] Losing government office, however, does not appear to affect personnel renewal, neither on the bivariate level, nor in the regression analysis. Overall, these patterns match the perspective that changes in the composition of the party executive reflect

---

[1] While we focus on party executive renewal between national elections here (which limits the number of observations particularly for NEOS) this also holds when calculating renewal rates between each party congress.

[2] Note that we do not draw substantive inferences from the positive effects of leadership change on personnel renewal found in Model 9, nor do we test a mediation hypothesis for leadership change in the main analysis. In our perspective, the two party-internal drivers of change should correlate but the specifics of this relationship are complex. While a designated party leader may at times demand (and thus trigger) further personnel changes in the party executive, both processes – leadership change and personnel changes in the party executive – will result from broader shifts in the party's internal power balance in other instances. It is even conceivable that changes in the composition of the party executive condition leadership change, as the former may be instrumental to the crumbling of an incumbent leader's support coalition at times. However, given our party-congress-based data, where changes in the party's top position and in the remainder of the party executive are all recorded at the same point in time, we cannot test such expectations empirically.

shifts in intra-party power. Also note that our data suggest a significant increase in renewal rates over our observation period (*r*=.29*) (Figure A1).

Figure A1: Over-time trend in party executive renewal (%).

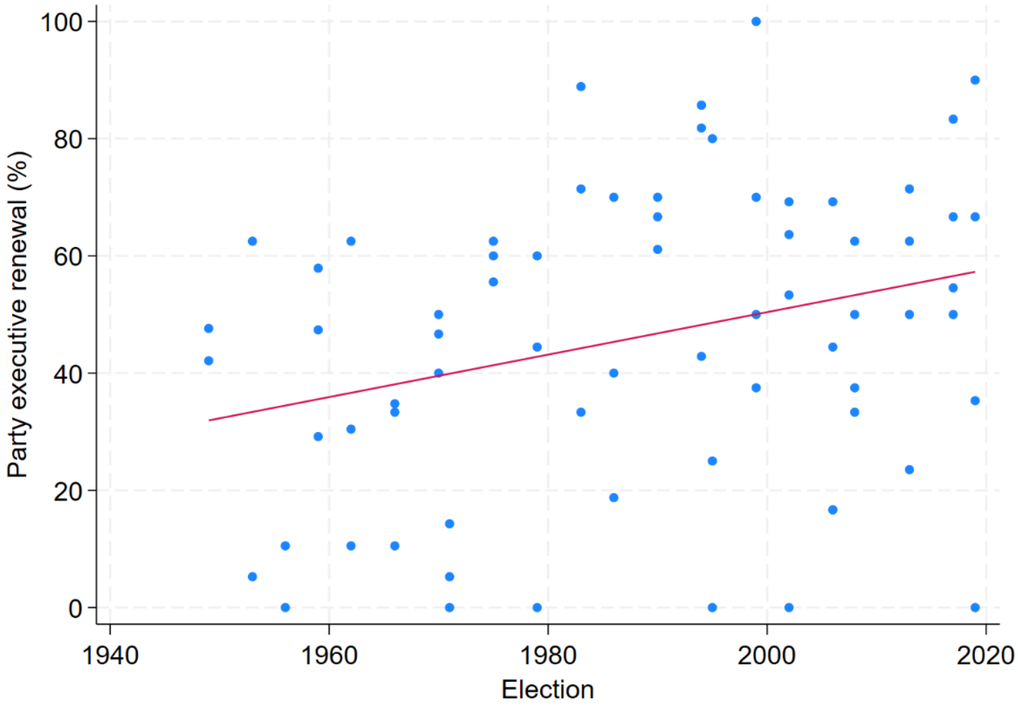

Our general assumption is that change in the personal composition of the party executive makes party policy change more likely than under stability in executive membership. Contrary to this expectation one might argue that group representatives may be replaced by like-minded successors. While intra-party groups may indeed select successors who are very similar to their predecessors regarding the respective group's main concerns, it is unlikely that this would be the case throughout the board and regarding all issue domains. In any case, replacement by like-minded successors would work against finding support for our hypotheses.

There is also the possibility of intra-person change of party executive members, either due to individual opinion conversion or opportunistic behavior of incumbents. While we cannot measure such change, we can highlight how it would affect the chances of finding support for our hypotheses. Depending on the nature of such change, it might either amplify or dampen the effects of personnel renewal in the party executive. Finally, although some replacements are most likely more consequential than others, we are unable to evaluate the impact of individual replacements.

Overview: Change in Issue Emphasis (Salience)

As outlined in the empirical-strategy section of the paper, we use change in issue emphasis as our measure for party policy change. We consider issue salience – as opposed to the party's ideological positioning – to be the more important aspect of intra-party competition over party policy. Typically, a party's positioning is not an overwhelming problem in most intra-party discussions. Policy positions are crucial to the party's identity (Harmel et al., 2018), they are rooted in ideological principles. More often than not, changing positions on specific issues will therefore incur costs in terms of external credibility and party-internal acceptance. In contrast, varying issue emphasis concerns innovation in the party program; it is about finding the right responses to a changing issue landscape and about fighting today's rather than yesterday's problems. However, a new cohort of party decision-makers may still move the party's position on the left—right (or other) ideological dimension(s) by changing the party's relative focus on different policy issues (Meyer and Wagner, 2019).

We construct our measure for change in issue emphasis by first calculating the salience of 20 policy issues areas in each party manifesto (welfare/services, taxes, labour, capital, regulation, security, social values, multiculturalism, education, environment, urban/rural, Europe, foreign policy, defense, constitutional issues, infrastructure, protest, ideology, government formation, residual category) (Dolezal et al., 2014) using the log measure of policy importance (Lowe et al., 2011). In a second step we record the change in emphasis on each of these issues relative to a party's previous electoral manifesto. Finally, we use the sum of absolute changes across all issues, divided by two, as our measure for the overall change in issue emphasis.

Considering other approaches to the measurement of issue emphasis in the extant literature, we additionally constructed measures for change in issue emphasis based on the method proposed by Prosser (2014) and based on the simple percentages of the manifesto covered by each issue as robustness checks. Note that our measure based on the Lowe et al. approach correlates highly with the Prosser-based measure (r=.89) and with a simpler aggregation of changes in the proportions of manifesto statements devoted to each issue category (r=.87). Regression models, indicating that our results are robust to using these alternative measurement approaches, are provided below.

Figure A2 further provides an overview of change in issue emphasis for each party and manifesto. To illustrate the correlation between these changes and personnel renewal for each party individually, the graphs additionally display the amount of change in leadership personnel. Notwithstanding other explanatory factors, these plots suggest that the connection

between personnel renewal and policy change is strongest for the ÖVP and weakest for the SPÖ, by tendency. However, a thorough investigation of these differences between parties would require additional data.

Finally, Table A3 displays the percentage of manifesto content (for all parties, across the entire observation period) attributed to each issue, to illustrate their overall salience in Austrian party competition.

Figure A2: Change in issue emphasis (log) and party executive renewal (%) by party.

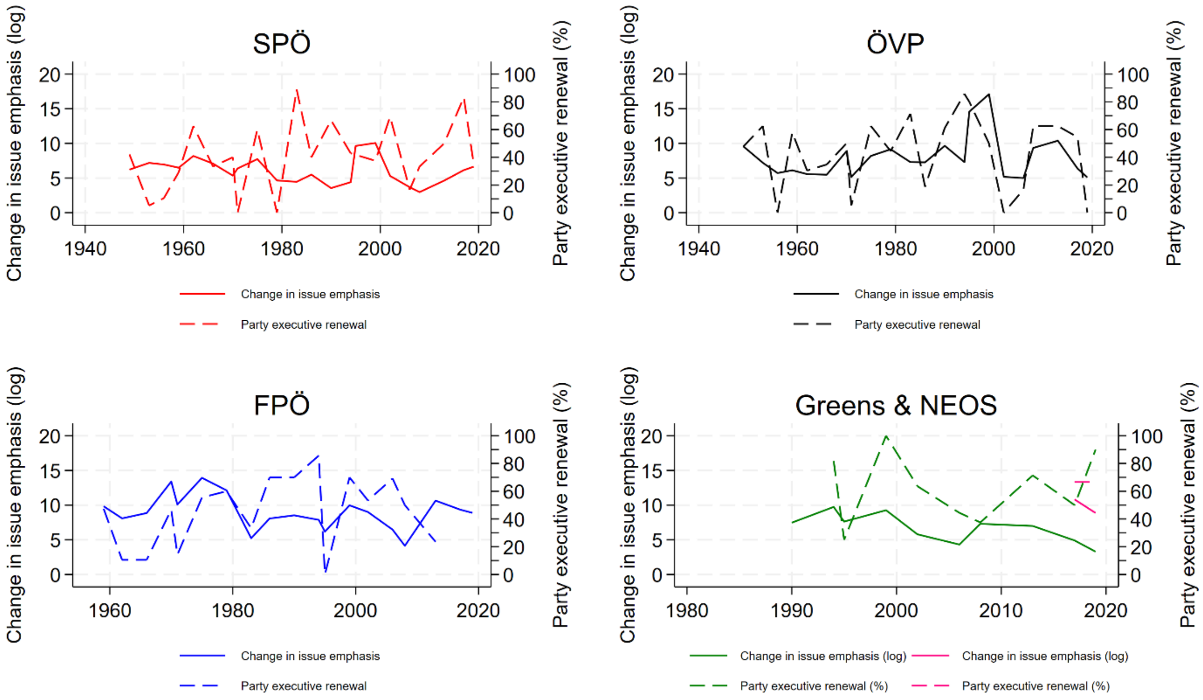

Table A3: Issue emphasis (salience) in Austrian party manifestos.

Issue area	Percentage of manifesto content (all parties, 1945-2019)
Welfare (services)	14.94
Education	13.22
Environment	9.15
Social values	7.86
Multiculturalism	6.56
Constitutional issues	6.43
Regulation	5.83
Labour	5.77
Taxes	5.76
Security	4.99
Capital (Enterprise)	4.11
Infrastructure	3.23
Europe	3.15
Foreign policy	2.63
Protest	2.17
Urban/Rural	2.07
Defense	1.43
Ideology	0.40
Government formation	0.31

Robustness: Alternative Control Variables

To test the robustness of our findings, we ran further regression models accounting for additional control variables. The first set of regressions (Table A4) indicates that our results on the drivers of change in a party's issue emphasis are robust to controlling for other potentially influential factors: the number of party executive members (Model 5), the length of the party's electoral manifesto (Model 6), electoral volatility (Model 7) and the time that has passed since the last election (Model 8). In the second set of OLS models (Table A5), we use party executive renewal as the dependent variable, analogous to Model 1 presented in the paper. In Models 9–11, we first include each of our predictors at a time for the sake of transparency. In Model 12 and Model 13, we then control for the size of the party executive and for the party's government/opposition status, as these factors might affect a party's renewal rate in the party executive. Findings are robust to using these alternative model specifications.

Table A4: OLS regression models on change in issue emphasis (salience); standard errors clustered by election.

	(5)	(6)	(7)	(8)
Party executive renewal (%)	0.0320**	0.0308*	0.0326**	0.0333*
	(0.0112)	(0.0120)	(0.0125)	(0.0156)
Leadership change	-0.388	-0.417	-0.368	-0.400
	(0.695)	(0.744)	(0.681)	(0.679)
Vote share change (%)	0.0238	0.0182	0.0231	0.0210
	(0.0541)	(0.0542)	(0.0519)	(0.0523)
Office loss	-1.497	-1.440	-1.525	-1.578
	(1.042)	(1.029)	(1.042)	(1.059)
Party in government	-0.299	-0.274	-0.313	-0.310
	(0.694)	(0.695)	(0.716)	(0.718)
Party executive members (N)	-0.0331			
	(0.0481)			
Manifesto statements (N)		0.0000545		
		(0.000232)		
Electoral volatility			0.0337	
			(0.120)	
Time since last election (days)				0.000284
				(0.00113)
Party FE	yes	yes	yes	yes
20y-period FE	yes	yes	yes	yes
Constant	5.957***	5.411***	5.202***	5.744***
	(0.999)	(0.865)	(1.236)	(1.331)
N	69	69	69	69
R^2	0.336	0.334	0.333	0.333

Standard errors in parentheses; + p<0.10, * p<0.05, ** p<0.01, *** p<0.001

Table A5: OLS regression models on party executive renewal; standard errors clustered by year.

	(9)	(10)	(11)	(12)	(13)
Leadership change	10.45+			5.618	4.624
	(5.545)			(5.649)	(5.340)
Vote share change (%)		-1.612***		-1.334**	-1.300**
		(0.354)		(0.457)	(0.450)
Office loss			-1.209	-2.547	3.827
			(8.414)	(9.434)	(10.32)
Party executive members (N)				0.0492	
				(0.454)	
Party in government					8.936
					(6.758)
Party FE	yes	yes	yes	yes	yes
20y-period FE	yes	yes	yes	yes	yes
Constant	27.45**	24.34*	27.24**	22.11+	14.11
	(8.496)	(10.12)	(9.890)	(12.86)	(10.61)
N	67	65	65	65	65
R^2	0.297	0.343	0.261	0.355	0.364

Standard errors in parentheses; + p<0.10, * p<0.05, ** p<0.01, *** p<0.001

Robustness: Alternative Operationalizations of Change in Issue Emphasis

In this section, we test whether the results of our statistical analysis are robust to using alternative operationalizations of change in issue emphasis. To this end we re-run our regression models on change in issue emphasis (Models 2–3), first using an emphasis change measure based on Prosser's salience approach (Prosser, 2014) (Models 14–16) as well as one using aggregated percentage changes (17–19) (Table A6).[3] Regression results using both measures essentially mirror those presented in the paper. Using the Prosser measure as dependent variable, we find a statistically significant positive effects of party executive renewal on change in issue emphasis, which is also comparable in size to the effects found in the main models (e.g. one standard deviation across the empirical range) (Models 14–15). Likewise, Model 16 indicates no statistically significant effects of competitive pressures on change in parties' issue focus. Using simple percentage changes as the dependent variable produces positive effects of personnel renewal as well. While similar in terms of effect size, this effect loses statistical significance in the full model (Model 18). Note, however, that there is a relatively pronounced correlation between the issue emphasis measure based on percentage change and time (r=.27, p<0.05) – potentially, because this approach is least suited

[3] In both variants we aggregate the absolute amount of change across all issues and divide this value by two, as we did for our primary measure of change in issue emphasis.

to account for differences in manifesto length, which has significantly increased over time. Amongst other things, we suspect this to bias our results when using the simple percentage change approach. Interestingly, though, we find a statistically significant effect of electoral performance in Model 19, which we expected, but which we did not find in the main models (H2a).

Table A6: OLS regression models on change in issue emphasis (salience) using measures based on Prosser (2014) (Models 14–16) and based on simple percentage changes (Models 17–19); standard errors clustered by election.

	DV: Change in issue emphasis (Prosser)			DV: Change in issue emphasis (%)		
	(14)	(15)	(16)	(17)	(18)	(19)
Party executive renewal (%)	0.00437*	0.00425*		0.0597*	0.0591	
	(0.00180)	(0.00180)		(0.0296)	(0.0375)	
Leadership change		-0.00174	0.0470		-2.766	-2.254
		(0.143)	(0.138)		(2.160)	(1.817)
Vote share change (%)		-0.00150	-0.00871		-0.288	-0.397*
		(0.0123)	(0.0123)		(0.200)	(0.199)
Office loss		-0.199	-0.250		-3.099	-3.124
		(0.193)	(0.182)		(3.355)	(3.168)
Party in government		-0.0659	-0.0610		-0.504	0.0175
		(0.146)	(0.146)		(2.408)	(2.068)
Party FE	yes	yes	yes	yes	yes	Yes
20y-period FE	yes	yes	yes	yes	yes	Yes
Constant	0.929***	0.951***	1.042***	22.66***	23.40***	24.35***
	(0.0907)	(0.173)	(0.170)	(1.543)	(2.983)	(2.921)
N	71	69	73	71	69	73
R^2	0.394	0.397	0.347	0.401	0.420	0.402

Standard errors in parentheses; + p<0.10, * p<0.05, ** p<0.01, *** p<0.001

Exploratory: Party Executive Renewal and Change in Policy Position

In this final section, we explore to what extent our findings on the drivers of change in issue emphasis also apply to change in policy position on a left–right ideological dimension. To this end, we first construct a measure for the left–right position of each manifesto, again based on coded AUTNES data (Dolezal et al., 2016; Müller et al., 2012), using the log scaling approach for policy positions proposed by (Lowe et al., 2011). We then use the absolute level of change in policy position compared to a party's previous manifesto as our dependent variable in Models 20–22 (Table A7).

In contrast to our findings on change in issue emphasis, party executive renewal does not affect change in policy position. However, a change in who is the party leader (another party-

internal driver of change) does lead to significantly larger policy shifts, which is not the case for change in parties' attention to policy issues. The results for other predictors of policy change are inconclusive, also when predicting parties' positional shifts. While change in issue emphasis and change in policy position are empirically related (based on our data we find a moderate correlation of *r*=.19 between them), our regression analyses thus suggest that these two forms of party policy change indeed follow different logics (see Harmel et al., 2018).

Table A7: OLS regression models on change in policy position (left–right) based on Lowe et al. (2011); standard errors clustered by election.

	(20)	(21)	(22)
Party executive renewal (%)	0.000800	0.000208	
	(0.000728)	(0.000887)	
Leadership change		0.121*	0.110*
		(0.0510)	(0.0483)
Vote share change (%)		0.00213	-0.000378
		(0.00533)	(0.00453)
Office loss		0.115	0.120
		(0.104)	(0.0986)
Party in government		-0.0109	-0.0236
		(0.0761)	(0.0814)
Party FE	yes	yes	yes
20y-period FE	yes	yes	yes
Constant	0.255**	0.296*	0.319**
	(0.0980)	(0.118)	(0.123)
N	71	69	73
R^2	0.0540	0.207	0.214

Standard errors in parentheses; + p<0.10, * p<0.05, ** p<0.01, *** p<0.001

References

Bolin N, Aylott N, Von dem Berge B, et al. (2017) Patterns of Intra-Party Democracy across the World. In: Scarrow SE, Webb PD, and Poguntke T (eds) *Organizing Political Parties: Representation, Participation, and Power*. Oxford: Oxford University Press, pp. 158–184.

Dolezal M, Ennser-Jedenastik L, Müller WC, et al. (2014) How Parties Compete for Votes: A Test of Saliency Theory. *European Journal of Political Research* 53(1): 57–76.

Dolezal M, Ennser-Jedenastik L, Müller WC, et al. (2016) Analyzing Manifestos in their Electoral Context A New Approach Applied to Austria, 2002–2008. *Political Science Research and Methods* 4(3): 641–650.

Harmel R, Tan AC, Janda K, et al. (2018) Manifestos and the "Two Faces" of Parties: Addressing Both Members and Voters with One Document. *Party Politics* 24(3): 278–288.

Lowe W, Benoit K, Mikhaylov S, et al. (2011) Scaling Policy Preferences from Coded Political Texts. *Legislative Studies Quarterly* 36(1): 123–155.

Meyer TM and Wagner M (2019) It Sounds Like They are Moving: Understanding and Modeling Emphasis-Based Policy Change. *Political Science Research and Methods* 7(4): 757–774.

Müller WC and Meth-Cohn D (1991) The Selection of Party Chairmen in Austria: A Study in Intra-Party Decision-Making. *European Journal of Political Research* 20(1): 39–65.

Müller WC, Philipp W and Steininger B (1992) Wie oligarchisch sind Österreichs Parteien? Eine empirische Analyse, 1945–1992. *Österreichische Zeitschrift für Politikwissenschaft* 21(2): 117–146.

Müller WC, Dolezal M, Ennser-Jedenastik L, et al. (2012) AUTNES Coding Instructions for Manifestos. Available at: http.//data.autnes.at/datadownload.htm.

Prosser C (2014) Building Policy Scales from Manifesto Data: A Referential Content Validity Approach. *Electoral Studies* 35: 88–101.
